# Effect of a short message service (SMS) intervention on adherence to a physiotherapist-prescribed home exercise program for people with knee osteoarthritis and obesity: protocol for the ADHERE randomised controlled trial

**DOI:** 10.1186/s12891-019-2801-z

**Published:** 2019-09-14

**Authors:** Rachel K. Nelligan, Rana S. Hinman, Jessica Kasza, Sarah Schwartz, Alexander Kimp, Lou Atkins, Kim L. Bennell

**Affiliations:** 1 Centre for Health, Exercise and Sports Medicine, Department of Physiotherapy, School of Health Sciences, The University of Melbourne, Melbourne, VIC Australia; 2 School of Public Health and Preventive Medicine, Monash University, Melbourne, VIC Australia; 3 Centre for Behaviour Change, University College London, London, UK

**Keywords:** Knee osteoarthritis, Exercise, Adherence, Behaviour change, SMS, Mobile phone, RCT, Trial

## Abstract

**Background:**

Knee osteoarthritis (OA) is a highly prevalent condition. People with knee OA often have other co-morbidities such as obesity. Exercise is advocated in all clinical guidelines for the management of knee OA. It is often undertaken as a home-based program, initially prescribed by a physiotherapist or other qualified health care provider. However, adherence to home-based exercise is often poor, limiting its ability to meaningfully change clinical symptoms of pain and/or physical function. While the efficacy of short message services (SMS) to promote adherence to a range of health behaviours has been demonstrated, its ability to promote home exercise adherence in people with knee OA has not been specifically evaluated. Hence, this trial is investigating whether the addition of an SMS intervention to support adherence to prescribed home-based exercise is more effective than no SMS on self-reported measures of exercise adherence.

**Methods:**

We are conducting a two-arm parallel-design, assessor-and participant-blinded randomised controlled trial (ADHERE) in people with knee OA and obesity. The trial is enrolling participants exiting from another randomised controlled trial, the TARGET trial, where participants are prescribed a 12-week home-based exercise program (either weight bearing functional exercise or non-weight bearing quadriceps strengthening exercise) for their knee by a physiotherapist and seen five times over the 12 weeks for monitoring and supervision. Following completion of outcome measures for the TARGET trial, participants are immediately enrolled into the ADHERE trial. Participants are asked to continue their prescribed home exercise program unsupervised three times a week for 24-weeks and are randomly allocated to receive a behaviour change theory-informed SMS intervention to support home exercise adherence or to have no SMS intervention. Outcomes are measured at baseline and 24-weeks. Primary outcomes are self-reported adherence measures. Secondary outcomes include self-reported measures of knee pain, physical function, quality-of-life, physical activity, self-efficacy, kinesiophobia, pain catastrophising, participant-perceived global change and an additional adherence measure.

**Discussion:**

Findings will provide new information into the potential of SMS to improve longer-term exercise adherence and ultimately enhance exercise outcomes in knee OA.

**Trial registration:**

Prospectively registered with the Australian New Zealand Clinical Trials Registry. Reference: ACTRN12617001243303

**Date/version:** August 2019/two

**Electronic supplementary material:**

The online version of this article (10.1186/s12891-019-2801-z) contains supplementary material, which is available to authorized users.

## Background

Osteoarthritis (OA) is a highly prevalent and disabling condition that most commonly affects the knee joint [1]. Many people with OA also have other chronic conditions including obesity, diabetes and heart disease [2]. OA is ranked as the 11th highest contributor to disability globally [3]. For the individual, OA causes pain, loss of function, psychological distress and reduced quality of life [4, 5]. For society, the economic costs, both direct and indirect, associated with OA are considerable, making OA a global public health problem [6]. With the prevalence of OA predicted to increase with population ageing and rising obesity rates [7], research into effective, scalable and inexpensive interventions to support conservative OA management are a priority.

As there is currently no cure for knee OA, empowering patients to self-manage their condition is vital [8]. The positive effects of exercise on pain and function in knee OA are widely reported [9, 10] and as such exercise is recommended as a core treatment in all clinical guidelines [8, 11–14]. Most commonly, exercise programs involve an initial period of supervision by a clinician, such as a physiotherapist, followed by unsupervised home-based exercise. Unfortunately, adherence to home-based exercise is often poor [15–17], particularly after clinician input ceases [16] and a wide variety of barriers may impact adherence such as pain, negative beliefs about exercise, life commitments [18]. This decline in exercise adherence, after input from a clinician ceases, is typically mirrored by a decline in the pain and physical function gains achieved during a more intensive period of exercise [17, 19, 20]. Improving longer-term adherence to prescribed home-based exercise may be a key strategy to enhance and maintain the symptomatic benefits of exercise and ultimately improve long-term patient outcomes in this chronic condition.

To date, there is uncertainty about how best to help people with knee OA adhere to prescribed home-based exercise. Interventions showing promise include ‘booster’ or ‘refresher’ sessions with a physiotherapist and behavioural graded exercise [21], which involves gradual increases in physical activity plus ‘booster’ sessions [22]. The benefits of clinician supervision on exercise adherence are well documented [23, 24] with a key benefit being the clinician’s ability to closely monitor and influence patient behaviour, in particular to intervene if waning adherence is identified. Such ongoing clinician involvement, however, may not be feasible or practical due to access challenges and/or cost. Further research into interventions promoting exercise adherence is needed. Such interventions should be easily scalable and accessible and, as recommended by the Medical Research Council, be developed systematically using theoretical frameworks [25].

The use of Short Messaging Services (SMS) to promote exercise adherence may be one solution. The effectiveness of SMS-based interventions to promote healthy behaviours relevant to OA such as physical activity, diet, and/or weight loss has been demonstrated in a range of settings and other conditions [26–29] with promising evidence highlighting that the immediate benefits of SMS on behaviour can be sustained after SMS contact ceases [30]. The use of SMS to improve adherence to home-based exercise has not yet been rigorously evaluated in people with knee OA. Only two small studies, a feasibility study and a pilot study, have investigated the use of mobile phone messaging [31, 32]. The pilot study [31] found no effect on adherence or functional outcomes with the addition of 12 video messages (Multi Media Messaging Service, MMS) delivered over 6-weeks. The messages were designed to be visual prompts to exercises and were not informed by behaviour change theory nor targeted to the individual. The feasibility study [32] assessed the effect of an educational booklet about OA and physical activity (delivered by mail), plus four weekly physical activity-promoting SMS, informed by Social Cognitive Theory. This study found significant positive effects on self-reported pain and exercise self-efficacy at 6 weeks. The intervention was well accepted by patients with 96% reporting they enjoyed receiving the messages and 88% finding them useful to promote physical activity. This feasibility study suggests that SMS has the potential to influence exercise outcomes (such as reduce pain and improve self-efficacy) in people with knee OA and is acceptable to patients. To investigate this further we developed a 24-week automated, semi-interactive SMS program to support adherence to prescribed home-based exercise for people with knee OA. The SMS program was developed rigorously applying behaviour change theory and is an inexpensive intervention, costing AUD $8 (approx.) per participant. The development of the SMS program is published elsewhere [33].

The primary aim of the ADHERE pragmatic randomised controlled trial (RCT) is to evaluate the effect of adding this 24-week SMS program to a prescribed home exercise program after cessation of clinician input. This trial will use a sample of participants exiting from another RCT, the TARGET trial, where participants are prescribed a home exercise program [34].

Our primary hypothesis is that adherence to the prescribed, unsupervised home exercise program will be higher at 24-weeks in the group receiving the SMS intervention compared to the group receiving no SMS contact. Our secondary hypothesis is that improvements in other outcomes such as measures of pain, function, health-related quality-of-life, global change, and another measure of exercise adherence will be greater in the SMS group compared to the no contact control group.

## Methods/design

### Trial design

**Fig. 1:**
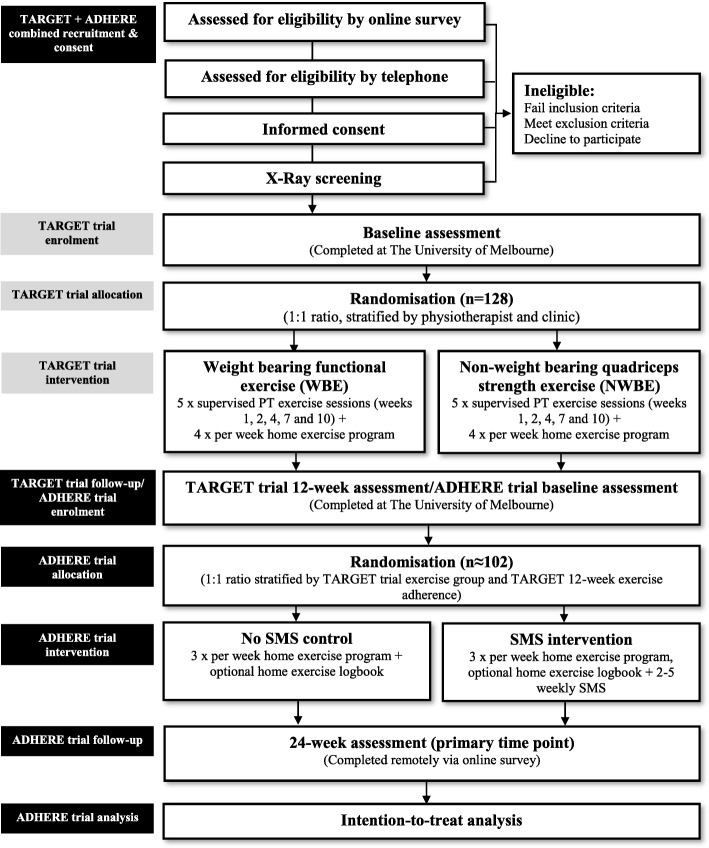
Flow diagram of TARGET and ADHERE trial procedures

This trial is a parallel-design 2-arm, assessor- and participant-blinded randomised controlled trial. Outcomes are assessed at baseline and 24 weeks after baseline. This protocol complies with the SPIRIT guidelines [35] with reporting following both CONSORT [36–38] and TIDieR guidelines [39]. Figure 1 outlines trial phases.

### Study setting

This trial proceeds the TARGET randomised controlled trial [34] which is comparing the effects of two different 12-week physiotherapist-prescribed home exercise programs on knee pain and physical function in individuals with knee OA and obesity. In the TARGET trial, participants visit a physiotherapist five times over 12 weeks to be prescribed either i) a weight bearing functional exercise program, or ii) a non-weight bearing quadriceps strengthening exercise program to be completed four times per week as a home program. Each exercise program includes five lower limb exercises which use equipment (ankle weights, resistance exercise band, exercise step, foam matting) and/or body weight for exercise progressions. Participants are taught by the physiotherapist how to perform the exercises, how to modify their program to maintain the exercise challenge set by the physiotherapist and how to manage knee symptom aggravation as required. Participants receive paper-based instructions of each exercise from their physiotherapist and an optional exercise logbook to record their exercise practice. Participants are instructed by their physiotherapist, at the final physiotherapy session (week 10/12 of the TARGET trial) and by the study coordinator, at TARGET completion/ADHERE enrolment, to continue their prescribed home exercise program three times per week, for an additional 24-weeks.

### Participants

This trial utilises participants completing the TARGET trial (*n* = 128) [34]. In the TARGET trial, participants are recruited from the community in Melbourne Australia and are aged ≥50 years, have painful medial knee OA and are obese (defined as body mass index (BMI) 30 kg/m^2^ and over). Table 1 summarises TARGET trial eligibility criteria. All participants who enrolled in the TARGET trial are eligible for the ADHERE trial. However, only those who complete their TARGET trial 12-week assessment and do not withdraw at this timepoint are enrolled into ADHERE. All participants provide written informed consent to participate at TARGET trial enrolment. Ethics approval has been obtained from the Human Research Ethics Committee of University of Melbourne (HREC No. 1544919).

**Table 1:** TARGET trial inclusion/exclusion criteria

Inclusion criteria	Exclusion criteria
Aged ≥50 years	Lateral joint space narrowing ≥ medial joint space narrowing on x-ray
Report knee pain on most days of the past month	Knee surgery/joint injection in past 6 months or planned surgery in the next 9 months
Suffered knee pain for 3 months or more	Current or past (4 weeks) oral corticosteroids
Report a minimum average overall pain score of 4 on an 11-point numeric rating scale over the previous week	Systemic arthritic conditions
Demonstrate tibiofemoral osteophytes on x-ray	Past knee fracture or malignancy
Obesity (BMI = 30 kg/m^2^ and over)	Past hip/knee joint replacement/tibial osteotomy
Have a mobile phone that has text messaging functionality and be happy to receive text message reminders if required during the study	Other condition currently affecting lower limb function
	Participation in knee strengthening or neuromuscular/functional exercise in past 6 months or planning to start exercise in next 9 months
	Unable to walk unaided
	Unable to commit to study requirements

*BMI* Body Mass Index

### Procedures

Baseline assessments are conducted at the Department of Physiotherapy, The University of Melbourne via online surveys or remotely via online surveys if participants are unable to attend. Follow-up assessments are completed remotely via online surveys. All data will be de-identified and stored in password protected computer folders. The final trial dataset will be accessible to all authors. In cases where both knees are equally symptomatic, the right knee will be selected as the focus of evaluation.

### Randomization and blinding

On completion of the TARGET trial final assessment (ADHERE trial baseline), participants undergo 1:1 randomisation into one of two groups: i) no SMS; or ii) SMS intervention. Computer-generated randomisation has been prepared by our study biostatistician (JK) and is conducted by permuted blocks of sizes 6 to 12, stratified by exercise group (based on TARGET intervention received, either weight bearing functional exercise or non-weight bearing quadriceps strengthening) and by baseline home exercise adherence in response to the question “In the past week, how many days did you do your home exercises?” with maximum number of days being 4; dichotomised into 0–1 = lower adherence and 2–4 to higher adherence).

To conceal allocation, the randomisation schedule is accessed via a password-protected computer program by a researcher not involved in participant recruitment or assessment. Participants are blinded to study groups, informed at TARGET trial enrolment, participation is for 9 months, with the initial 3 months (TARGET trial) comparing the effects of two different exercise programs and the following 6 months (ADHERE trial) investigating strategies aimed at helping them stick to a prescribed exercise program, which may include a log book and encouraging text messages. To avoid influencing exercise adherence behaviour participants are not informed of the two separate studies, of re-randomisation into this trial or exact intervention details. The statistician is blinded to group allocation, which will be revealed upon completion of the statistical analyses.

### Interventions

#### No SMS

Participants in the control group are asked to continue the home-based exercise program prescribed to them in the TARGET trial, three times weekly for 24 weeks. Participants are not restricted from taking medication or pursuing other knee interventions during the 24-weeks. This information will be collected at the 24-week timepoint, via online survey.

#### SMS intervention

In addition to continuing the home-based exercise program prescribed in the TARGET trial, participants in this group receive a SMS intervention to support adherence. The SMS intervention is a 24-week automated SMS program delivered via mobile phone designed to support adherence to prescribed home-based exercise.

The development of the SMS intervention is reported elsewhere [33]. Briefly, this intervention was designed applying the Behaviour Change Wheel (BCW) framework which is a synthesis of 19 models of behaviour providing a transparent and systematic framework for the development of behaviour change interventions [40, 41]. The BCW uses the COM-B model to describe the influences of behaviour using three interacting categories; i) capability (e.g. skills and knowledge), ii) opportunity (e.g. resources and social cues) and iii) motivation (e.g. intentions and desires). To provide greater detail the COM-B categories can be further divided into the 14 domains of the Theoretical Domains Framework (TDF) [42]. The intervention content was informed by our previous research which identified key barriers and facilitators to exercise adherence in knee/hip OA mapped to the TDF and the COM-B model of the BCW [18]. Behaviour change techniques (BCTs) which linked to the COM-B/TDF domains and each barrier/facilitator, were then selected from the Behaviour Change Technique Taxonomy (BCTTv1) [43] as recommended in the BCW. The selected BCTs were then used to construct the SMS interventions messages.

For the SMS intervention, participants receive up to five text messages weekly. Message frequency reduces over the 24-week intervention, as recommended in the literature, to lessen participant burden [44]. Message length ranges from 105 to 420 characters. Each week (weeks 1–8) to each fortnight (weeks 9–24) participants receive a message asking them to self-report the number of home exercise sessions completed in the previous week. Participants who report low adherence (≤2 exercise session/week) then receive a message prompting them to select a barrier from a predetermined list (forgot, too tired, knee hurts so can’t exercise, worried exercise is causing pain, exercise isn’t helping, boring, lack of time, life stress, and none above apply to me) which best explains why they were unable to complete their exercises as prescribed (three times in the previous week). Participant barrier selection then triggers a BCT message providing a suggestion tailored to help address the selected barrier. Additional file 1 lists the BCTs used to address each barrier. Program automation is designed to prevent the same message being sent if the same barrier is selected more than once. Participants who report being adherent (≥3 exercise session/week) receive a positive reinforcement message encouraging continued completion of the home exercises three times each week with program automation ensuring a different message is received each time. Participants also receive regular motivational SMS (twice weekly initially and reducing to once fortnightly by 24-weeks) containing BCT suggestions linked to exercise facilitators. Additional file 2 lists the exercise facilitators targeted in the intervention and the BCTs which were converted into SMS to address them. Furthermore, to enhance engagement, participants receive special occasion messages (e.g. birthday, Christmas).

After randomisation, each participant is guided by a researcher through a three-message practice sequence to assist in replying to messages and are informed that they can opt out of the SMS intervention at any time by sending the word “stop” to the SMS program number. This practice session is in person at the University of Melbourne or remotely via email if the participant is unable to attend baseline assessment at the university. Participants are not restricted from taking medication or pursuing other knee interventions during the 24-weeks.

### Outcomes

Outcome measures and time points are summarised in Table 2. The primary time point is at 24 weeks.

**Table 2:**
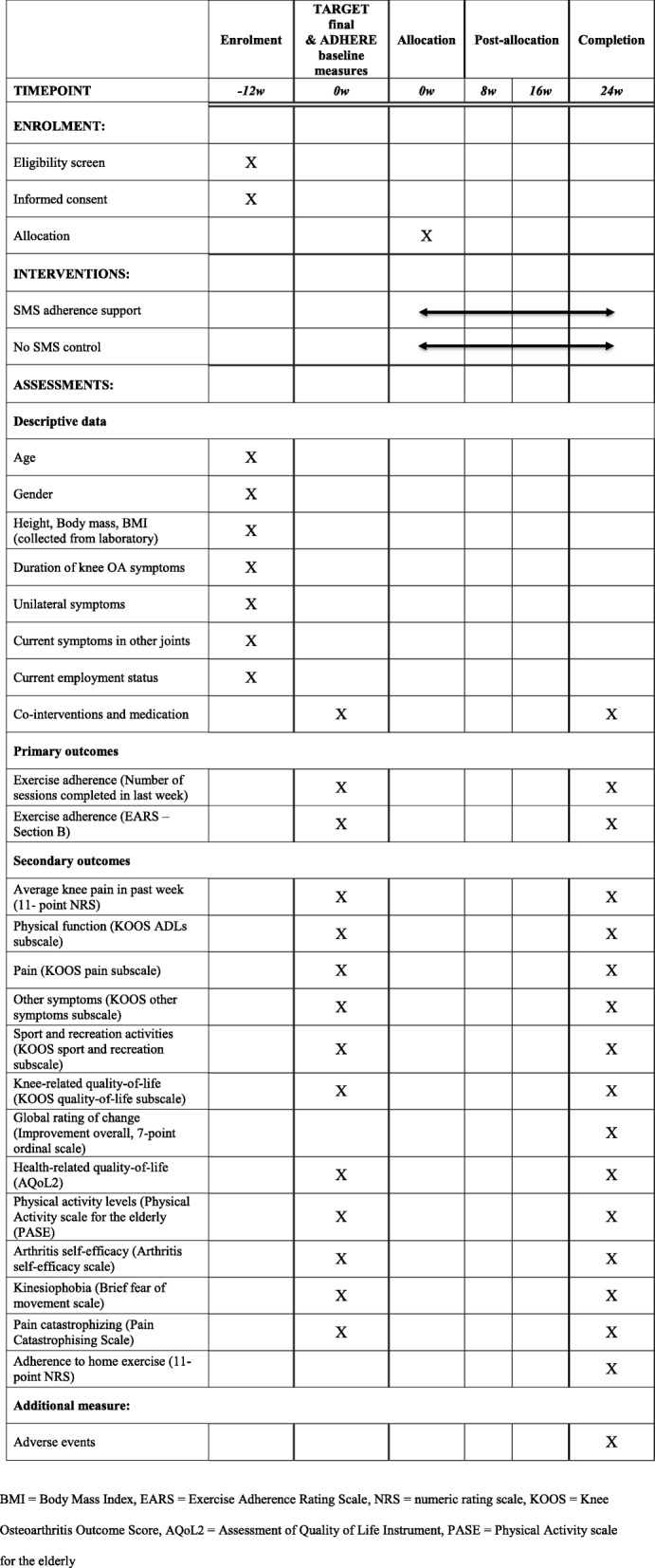
Schedule of enrolment, intervention, and assessments

*BMI* Body Mass Index, *EARS* Exercise Adherence Rating Scale, *NRS* numeric rating scale, *KOOS* Knee Osteoarthritis Outcome Score, *AQoL2* Assessment of Quality of Life Instrument, *PASE* Physical Activity scale for the elderly

#### Descriptive data

Age, gender, duration of knee OA symptoms, previous treatments, problems in other joints, and measures of height and body mass are obtained from the TARGET trial baseline assessment. Height and weight measures are used to calculate BMI. Baseline number of exercise sessions (the number of exercise sessions completed in the past week) and baseline exercise adherence using the Exercise Adherence Rating Scale (EARS) Section B [45] will both be taken from the final TARGET trial time-point.

#### Primary outcomes

Primary outcomes are two self-reported measures of adherence to prescribed home exercise collected at 24 weeks:

##### Exercise adherence rating scale (EARS) section B

This questionnaire measures adherence to prescribed home exercise using 6-items scored on a 5-point scale with terminal descriptors of ‘strongly agree’ to ‘strongly disagree’. Items 1, 4 and 6 are reverse scored, resulting in a possible total score of between 0 and 24. A higher score indicates better adherence. This measure has acceptable internal consistency and high test-retest reliability [45].

##### Self-reported number of home exercise sessions completed in week 23

Participants are asked “In the PAST WEEK, how many days did you do your home exercises?” Responses range from 0 days to 3 days.

#### Secondary outcome measures

A range of self-reported secondary outcomes are measured at baseline and 24 weeks, unless indicated otherwise.

##### Self-rated adherence to the home exercise program (24-week only)

Participant adherence to the home exercise program across the 24 weeks is based on their agreement to the statement “I have been doing my exercise sessions 3 times each week as recommended”. Responses will be collected using an 11-point scale with terminal descriptors “strongly disagree” =1 to “strongly agree” =10.

##### Overall knee pain

Average overall knee pain in the past week is self-assessed using a 11-point numeric rating scale (NRS) [46] with terminal descriptors of ‘no pain’ (score 0) and ‘extreme pain’ (score 10).

##### Pain, other symptoms, function in daily living, function in sport and recreation and knee related quality of life in the last week

The five subscales of the Knee Injury and Osteoarthritis Outcome Score (KOOS) are being measured [47]. These subscales are Pain (9-items), Other symptoms (7-items), Function in daily living (ADL) (17-items), Function in sport and recreation (5-items) and Knee related quality of life QOL (4-items). Responses are provided on a 5-point scale and range from 0 to 100 where higher scores represent better outcomes.

##### Health-related quality of life

The AQoL questionnaire (version AQoL-II) [48] is measuring health related quality of life using 20-items covering six dimensions, including independent living, social relationships, physical senses, coping, pain and psychological wellbeing. Responses are provided on a 5-point scale − 0.04 to 1.00 with 1 represents full health-related quality of life.

##### Self-efficacy

The Arthritis Self-Efficacy Scale [49] is measuring arthritis specific self-efficacy. The three subscales will be collected; self-efficacy for managing pain (5-items), physical function self-efficacy (9-items) and other symptoms self-efficacy (6-items). Responses are provided on a 10-point scale and range from 0 to 10 where higher scores represent greater self-efficacy.

##### Kinesiophobia

The Brief Fear of Movement Scale for OA, a 6-item scale, assesses activity avoidance due to pain-related fear of movement [50]. Responses are provided on a 4-point scale and range from 6 to 24 where higher scores represent more fear.

##### Pain Catastrophising

The Pain Catastrophising Scale [51] measures level of pain catastrophising using 13-items covering three dimensions including rumination (4-items), magnification (3-items), and helplessness (6-items). Responses are provided on a 5-point scale and range from 0 to 52 where higher scores representing greater catastrophising.

##### Physical activity

The Physical Activity Scale for the Elderly [51] assess physical activity, over the previous week, using 10-items. Scoring is calculated using the frequency, duration, and intensity level of a range of activities typically chosen by older adults and range from 0 to > 400 where higher scores representing greater levels of physical activity.

##### Participant-perceived response to treatment (24 week only)

Participants rate their perceived change, since baseline, in their condition overall on a 7-point scale with terminal descriptors of “much worse” to “much better”. Participants who report “moderately better” and above are classified as improved [52].

Other measures will include:

##### Adverse events

Any problem that participants believe was caused by the advice received as part of the study and required them to seek treatment/take medications, and/or interfered with function for two or more days are recorded via questionnaire at 24 weeks.

##### Co-interventions

Medications and other treatments for knee OA are recorded at 24 weeks using a custom-developed survey. This survey records the frequency of use of a range of pain and arthritis medications and co-interventions over the past 6 months.

### Data analysis, monitoring and auditing

#### Sample size calculation

The ADHERE trial includes eligible participants exiting from the TARGET trial (*n* = 128 total initial sample for TARGET). We conservatively estimate that 102 participants (80% of the 128 TARGET enrollees) will be randomised into the ADHERE trial, and that of those 102 participants, 82 (80%) will be retained in ADHERE until week 24. With 40 participants per group, we will have 83% power to detect an effect size of 0.6 with two-sided significance level of 0.05. This assumes a correlation between baseline home exercise adherence (taken from TARGET trial final time-point) and adherence outcomes at 24 weeks of 0.4, based on data from our previous trials [53–55] and including baseline adherence in the regression model as a covariate. This calculation assumes that the effect size of interest is the same for both primary outcomes and therefore applies to both/either.

#### Data analysis

Analyses comparing the two groups will be performed by the statistician in a blinded fashion using all available data from all randomised participants according to the intention-to-treat principle. Baseline characteristics of participants will be summarised as appropriate (means and standard deviations for continuous variables that appear to be approximately symmetrically distributed, medians and interquartile ranges for other continuous variables, counts and percentages for categorical variables) and will be inspected to assess baseline comparability of treatment groups. For continuous outcomes, differences at 24-weeks or in change (baseline minus follow-up) will be compared between groups using linear regression models adjusted for baseline measures and the stratifying variables of TARGET exercise group and dichotomised baseline adherence (number of exercise sessions completed in the past week represented as low versus high adherence). Model assumptions will be assessed using standard diagnostic plots. For binary outcomes, differences between groups will be compared using relative risks, calculated from logistic regression models including terms for TARGET exercise group and dichotomous baseline adherence [56]. Should the amount of missing data for an outcome be such that imputation is required (i.e. > 5%), multiple imputation will be conducted and the method reported.

#### Monitoring

The trial coordinator and lead investigators meet fortnightly to monitor adverse events, any issues relating to the trial, review recruitment and trial progress.

#### Dissemination plans

Findings of this trial will be presented at conferences, published in a peer-reviewed journal and a lay summary of findings provided to all participants. In addition, dissemination of findings will be through research networks including the Centre for Health, Exercise and Sports Medicine, and the National Health and Medical Research Council Centre for Research Excellence in Translational Research in Musculoskeletal Pain.

## Discussion

This RCT is investigating if a behaviour change theory-informed SMS intervention that addresses key barriers and facilitators to exercise adherence in people with knee OA, delivered once clinician involvement ceases, can improve adherence to a prescribed home-based exercise program over 24-weeks when compared to a no contact control. This study will provide insight into the effectiveness of SMS technology to promote adherence in the knee OA population and if effective will be an easily scalable, cheap and accessible intervention for people with knee OA including those in regional and remote areas.

## Additional files

Additional file 1: Exercise barriers and the BCTs which were converted into individual SMS to address each barrier. Demonstrates the application of the BCW framework using COM-B categories, TDF domains and intervention functions. (PDF 201 kb)

Additional file 2: Exercise facilitators and the BCTs which were converted into individual SMS to address each facilitator. Demonstrates the application of the BCW framework using COM-B categories, TDF domains and intervention functions. (PDF 202 kb)

## Data Availability

The SMS programs behaviour change message library will be available by request on a case-by-case basis at the discretion of the corresponding author.

## Abbreviations

BCT: Behaviour Change Technique
BCW: Behaviour Change Wheel
SMS: Short Messaging Service
