# Supplementary material for: Effect of a short message service (SMS) intervention on adherence to a physiotherapist-prescribed home exercise program for people with knee osteoarthritis and obesity: protocol for the ADHERE randomised controlled trial
Source: BMC Musculoskelet Disord. 2019 Sep 14;20:428. doi: 10.1186/s12891-019-2801-z (PMC6745064; doi:10.1186/s12891-019-2801-z)
Supplement: Supplementary file 1 — Exercise barriers and the BCTs which were converted into individual SMS to address each barrier. Demonstrates the application of the BCW framework using COM-B categories, TDF domains and intervention functions. (PDF 201 kb) [file 12891_2019_2801_MOESM1_ESM.pdf]

Additional file 1

Exercise barriers and the BCTs which were converted into individual SMS to address each barrier (mapped following the BCW framework using COM-B categories, TDF domains and intervention functions)

| **Barrier** | **COM-B category** | **TDF Domain** | **Relevant Intervention function** | **BCT used within the SMS intervention** |
|---|---|---|---|---|
| 1. Forgetfulness | Psychological capability | 10. Memory, attention and decision processes | Training | 8.3 Habit formation |
| | | | Environmental restructuring | 7.1 Prompts/cues |
| | | | Enablement | 3.1 Social support (unspecified) |
| | | | | 10.9 Self-reward |
| | | | | 1.2 Problem Solving |
| | | | | 1.4 Action Planning |
| 2. Too tired | Psychological capability<br><br>Reflective motivation* | 10. Memory, attention and decision processes<br><br>8. Intentions | Education* | 5.1 Information about health consequences * |
| | | | Persuasion* | - |
| | | | Training | - |
| | | | Environmental restructuring | - |
| | | | Enablement | 1.4 Action planning |
| | | | | 15.4 Self talk |
| | | | | 15.1 Verbal persuasion about capability |
| | | | | 1.2 Problem Solving |
| | | | | 8.7 Graded tasks |
| 3. Knee pain limiting perceived ability to exercise | Reflective motivation | 4. Beliefs about Capabilities | Education | 5.1 Information about health consequences |
| | | | Persuasion | - |
| | | | Enablement | 8.7 Graded tasks |
| | | | | 12.4 Distraction |
| | | | | 15.4 Self talk |
| | | | | 1.4 Action Planning |
| | | | | 1.2 Problem Solving |
| 4. Concern exercise (causing) pain + Fear of damaging knee further | Reflective motivation | 6. Beliefs about consequences | Persuasion | 5.1 Information about health consequences |
| | | | Enablement | 1.2 Problem Solving |
| | | | | 1.4 Action planning |
| 5. Lack of improvement with exercises | Automatic motivation | 7. Reinforcement | Training | 2.4 Self-monitoring outcome(s) of behaviour |
| | | | | 2.2 Feedback on behaviour |

| | | | | |
|---|---|---|---|---|
| | | | | 4.4 Behavioural experiments |
| | | | | 4.1 Instruction on how to perform the behaviour |
| | | | Environmental restructuring | - |
| 6. Lack of enjoyment in exercise + Boredom with exercise | Automatic motivation | 13. Emotion | Persuasion | 5.1 Information about health consequences |
| | | | Enablement | 12.4 Distraction |
| | | | | 15.4 Self talk |
| | | | | 10.9 Self-reward |
| | | | | 12.1 Restructuring the physical environment |
| | | | | 1.2 Problem Solving |
| | | | | 1.4 Action Planning |
| | | | | 3.1 Social support (unspecified) |
| 7. Conflict with routines + Lack of time | Reflective motivation | 11. Environmental context and resources | Training | 4.1 Instruction on how to perform the behaviour |
| | | | Environmental restructuring | - |
| | | | Enablement | 3.1 Social support – unspecified |
| | | | | 1.2 Problem solving |
| | | | | 1.4 Action Planning |
| | | | | 15.3 Focus on past success |
| | | | | 1.3 Goal setting (outcome) |
| 8. Family commitments + Increased social strain + Life events | Social Opportunity | 12. Social influences | Environmental restructuring | - |
| | | | Enablement | 1.4 Action Planning |
| | | | | 2.4 Self-monitoring of outcomes of behaviour |
| | | | | 15.1 Verbal persuasion about capability |
| | | | | 1.2 Problem solving |
| | | | | 15.4 Self talk |
| | | | | 15.3 Focus on past success |
| | | | | 1.1 Goal setting (behaviour) |

BCT= Behaviour change technique

BCW=Behaviour Change Wheel

Dash (-) indicates no BCT messages used in final SMS library from this intervention function
